# *Who are we missing?* Non-participation in an Internet intervention trial for depression and anxiety in adolescents

**DOI:** 10.1007/s00787-012-0295-4

**Published:** 2012-06-27

**Authors:** Willemijn Hoek, Floor Aarts, Josien Schuurmans, Pim Cuijpers

**Affiliations:** Department of Clinical Psychology, Faculty of Psychology and Education, EMGO+ Institute, VU University, Van der Boechorststraat 1, 1081 BT Amsterdam, The Netherlands

Dear editor,

Providing the high incidence of internalizing problems in adolescence and given that most adolescent health problems are caused by preventable behaviors, there is a critical need for healthcare for this population [1]. Yet, adolescents use the health care system less compared with any other age groups [1]. Providing interventions that can overcome help-seeking barriers and consequently include a low threshold for entry may facilitate treatment consideration and participation [2]. At the VU University, we conducted a randomized controlled trial (RCT) to investigate the effects of an Internet-based guided self-help intervention for adolescents reporting mild-to-moderate symptoms of depression and/or anxiety as compared to a wait-list control group [3]. We hypothesized to perceive less non-response than traditional interventions, since the Internet has low threshold acceptability and reduces objections like lack of willingness to talk to a stranger about personal problems and fear of stigma, and the self-help format of the intervention leaves patients to work at their own pace and in their own time [3]. However, despite the use of various recruitment strategies, only a limited amount of adolescents signed up for the study or chose to participate.

**Fig. 1:**
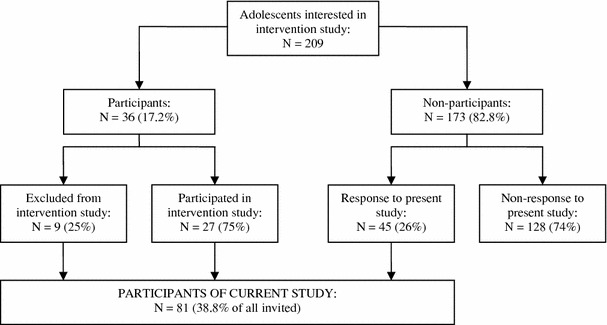
Flow chart of adolescent recruitment in the intervention study and in the present study

We examined non-participation, and consequently the generalizability of findings, for our Internet intervention trial for symptoms of depression and/or anxiety. Self-report data were collected from adolescents who decided to participate in the intervention study (participants) and adolescents who signed in on the website but eventually decided not to participate (non-participants). Over the 15-month recruitment period, 209 adolescents signed in on the website. 36 adolescents and their parents (17.2 %) forming our participants group gave informed consent. Non-participants were contacted by email and requested to complete an online self-report questionnaire on reasons for non-participation in the intervention study, and on socio-demographics, intervention expectations (one item from the CEQ8 [4]) and mental health determinants (depressive symptoms, anxiety symptoms and suicidal intention, as measured by the CESD [5], HADS-A [6] and BDI [7], respectively) identical to the baseline measure for participants. 45 (26.0 %) of the 173 adolescents who were contacted completed a questionnaire. Figure 1 presents the flow chart of adolescent recruitment.

Results indicate that immigrants and males were fairly underrepresented among adolescents who were interested in the intervention trial. No demographic differences were found between groups and comparison of the associations between socio-demographics and mental health factors for participants and non-participants did not reveal any significant interaction effects.

To assess the role of various determinants that were presumed by the investigators to influence the decision not to participate, a 6-point Likert scale from 0 (no role) to 5 (major role) was used, as well as one open question on why the adolescent did decide not to participate in the study. Regarding presumed determinants, parental permission played the most important role in the decision to decline participation, followed by having considered different treatments, study randomization with risk of being randomized to a wait-list condition, higher attractiveness of chatting online than self-help, high time investment, participating in questionnaires and diagnostic interview, (non)attractiveness of self-help format, a perceived lack of support from parents/friends/family, and appearance and lay out of brochure and website (See Table 1). The answers to the open-response question have been abstracted into main reasons for non-participation, and most often reported was asking for parental permission (36 %). Other reasons for not participating were looking for or receiving other treatment (13.3 %), time investment (11.1 %), participating in diagnostic interview (8.9 %), not meeting inclusion criteria because of age requirements (*n* = 3) or symptom severity (*n* = 1) (8.9 %), a significant decrease or absence of internalizing symptoms (8.9 %), not knowing what to expect (4.4 %) and forgetting about it (4.4 %).

**Table 1:** The role of determinants in the decision not to participate in the intervention study reported on a 6-point Likert scale from 0 (no role) to 5 (major role)

Reasons	Mean	SD	Score 0–3^a^ (%)	Score 4–5^b^ (%)
(1) Asking my parents for permission to participate in the study poses a problem for me	2.2	2.3	60.0	40.0
(2) I have considered other types of treatment than the present self-help course, to deal with my depressive and/or anxiety symptoms	2.2	1.9	71.1	28.9
(3) I do not want to run the risk of being randomized to the wait-list condition, and having to wait for 4 months before I can start the course	1.9	1.7	82.2	17.8
(4) Chatting online is more appealing to me than a self-help course delivered through the Internet	1.8	1.7	84.5	15.5
(5) The Internet intervention is too time consuming for me	1.7	1.9	75.6	24.4
(6) I do not feel like filling in questionnaires and participating in an interview	1.5	1.6	84.4	15.6
(7) A self-help course does not suit me	1.3	1.3	93.4	6.6
(8) I experience a lack of support from my parents/friends/family for participating in the course	1.2	1.7	86.7	13.3
(9) The appearance, lay out, and writing style of/on the information brochure and website does not appeal to me	1.1	1.0	97.8	2.2

^a^Determinant plays no role or a small role in the decision not to participate

^b^Determinant plays a major role in the decision not to participate

For each determinant that was assumed to influence the decision not to participate, associations with mental health factors were examined. Linear regression analyses showed that depression was significantly related to perceived lack of support from parents/friends/family (*p* < 0.001) and requirements for parental permission (*p* < 0.005) as barriers for participation, showing that the more these determinants were perceived to play a role in the decision not to participate, the more depressive symptoms were encountered. For anxiety, the more non-participants reported that looking for other treatments (*p* = 0.037), a lack of support from parents/friends/family (*p* = 0.006), and requirements for parental permission (*p* = 0.016) were reasons for non-participation, the more symptoms were encountered. Suicidal intentions were associated with a lack of support from parents/friends/family (*p* = 0.003), requirements for parental permission (*p* = 0.018), and higher attractiveness of chatting online than self-help (*p* = 0.013) as barriers for participation. For each of the three main reasons (i.e., as reported by more than 10 % of non-participants) for non-participation as measured by the open-response question, it was investigated whether reporting that particular reason as the main reason for non-participation (yes, no) was predictive of mental health outcomes. Regression analyses revealed that non-participants who declined because of parental permission requirements reported more depressive symptoms than other non-participants (*M* = 43.67, SD = 7.48 versus *M* = 27.50, SD = 13.96), *p* = 0.005, and more anxiety symptoms (*M* = 13.63, SD = 2.20 versus *M* = 8.79, SD = 5.58), *p* = 0.030. Moreover, they reported more suicidal intentions than non-participants whose reason for non-participation were not related to requirements for parental permission (*M* = 1.78, SD = 0.83 versus *M* = 0.86, SD = 1.10), *p* = 0.003.

Although we sought to construct a low-threshold intervention, we inadvertently constructed a process that was unfeasible and difficult for adolescents. To conclude, this study raises a fundamental question about the external validity of findings from Internet-based intervention efficacy trials for depression and anxiety in adolescents. Research requirements for obtaining parental consent particularly hindered participation rates and may constrain the opportunity to test effects of interventions. Our findings suggest that we may have excluded adolescents who are at particularly high risk for internalizing symptoms though not receiving any treatment because of a lack of support from parents/friends/family and perceived parental consent barriers, which poses a risk to the adolescent. Overall, it is worrying that we are presently not capable of reaching a greater number of adolescents with emotional difficulties in research.
